# Minimizing fungal disease deaths will allow the UNAIDS target of reducing annual AIDS deaths below 500 000 by 2020 to be realized

**DOI:** 10.1098/rstb.2015.0468

**Published:** 2016-12-05

**Authors:** David W. Denning

**Affiliations:** 1 Global Action Fund for Fungal Infections (GAFFI), Rue de l'Ancien-Port 14, 1211 Geneva 1, Geneva, Switzerland; 2 The National Aspergillosis Centre, University Hospital of South Manchester, The University of Manchester, Manchester Academic Health Science Centre, Manchester M23 9LT, UK

**Keywords:** *Aspergillus*, *Pneumocystis*, *Histoplasma*, *Cryptococcus*

## Abstract

Deaths from AIDS (1 500 000 in 2013) have been falling more slowly than anticipated with improved access to antiretroviral therapy. Opportunistic infections account for most AIDS-related mortality, with a median age of death in the mid-30s. About 360 000 (24%) of AIDS deaths are attributed to tuberculosis. Fungal infections deaths in AIDS were estimated at more than 700 000 deaths (47%) annually. Rapid diagnostic tools and antifungal agents are available for these diseases and would likely have a major impact in reducing deaths. Scenarios for reduction of avoidable deaths were constructed based on published outcomes of the real-life impact of diagnostics and generic antifungal drugs to 2020. Annual deaths could fall for cryptococcal disease by 70 000, *Pneumocystis* pneumonia by 162 500, disseminated histoplasmosis by 48 000 and chronic pulmonary aspergillosis by 33 500, with approximately 60% coverage of diagnostics and antifungal agents; a total of >1 000 000 lives saved over 5 years. If factored in with the 90–90–90 campaign rollout and its effect, AIDS deaths could fall to 426 000 annually by 2020, with further reductions possible with increased coverage. Action could and should be taken by donors, national and international public health agencies, NGOs and governments to achieve the UNAIDS mortality reduction target, by scaling up capability to detect and treat fungal disease in AIDS.

This article is part of the themed issue ‘Tackling emerging fungal threats to animal health, food security and ecosystem resilience’.

## 1. Introduction

Too many people die from AIDS, most of them adults in the prime of life. The majority of these deaths are attributable to opportunistic infections. As an example, across the world, HIV infection is second only to traffic accidents as a cause of death in adolescents [1]. Dying from AIDS greatly reduces family income, increasing poverty and inequality as well as the overall macroeconomic performance of high burden countries—Botswana, South Africa and Nigeria being recently studied examples [2–4].

In 2010, UNAIDS issued the aspirational target of zero AIDS deaths by 2015 [5]. Yet still 1 500 000 people died of AIDS in 2013 [6], a reduction of only 15% from 1 760 000 lives lost in 2010. Retention in care is a major factor [7] but late presentation with overwhelming infection is another. Major efforts to diagnose and treat HIV and tuberculosis (TB) co-infection have made an important impact, but still an estimated approximately 360 000 died from this in 2013 [8]—24% of the estimated total of 1 500 000 who died of AIDS. Efforts to treat hepatitis and HIV co-infection are accelerating, but very few HIV patients die of these dual infections although 500 000 are thought to die of the complications of hepatitis C worldwide [9].

A major focus on efforts to reduce deaths from fungal diseases complicating HIV infection could reduce AIDS deaths by more than 30%. The major fungal causes of death in AIDS are cryptococcal meningitis, *Pneumocystis* pneumonia (PCP), disseminated histoplasmosis (DH) and, probably, aspergillosis (chronic and invasive). Diagnostic tools and therapies for these entities are available but are either not available or not used in many high burden countries, Nigeria being one prominent example [10].

A straightforward, linear scenario prediction exercise from 2016 to 2020 was undertaken to estimate the relative impact of addressing fungal diseases to reduce mortality. In summary, the current slow downward trajectory of AIDS deaths was compared with additional, gradually increasing efforts to diagnose and treat cryptococcal disease, PCP, DH and chronic pulmonary aspergillosis (CPA) after TB, as well as the benefits of increased antiretroviral therapy (ART) coverage as per the 90–90–90 campaign programme.^1^ To ensure that the figures presented are conservative, it is assumed that ART retention on therapy is excellent and resistance is minimal, whereas over 20% of patients do not meet these assumptions in low- and middle-income countries (LMICs) [7–11] (see endnote 1).

## 2. Methodology

Recent global estimates of HIV-infected patients [1], annual AIDS deaths [1] and TB [8] were used as baseline data. Estimates of the annual incidence of cryptococcal disease, PCP, DH and CPA were derived, as explained in each section, using existing data on incidence rates. Current data on survival from these infections, if diagnosed and treated, were used to estimate mortality for each infection. It was assumed that patients only have one of these infections at presentation and that each patient only has one episode in their lifetime. The baseline assumptions are summarized in table 1. The number of TB patients reported with and without HIV infection in 2013, and deaths with smear-positive and smear-negative TB, are shown in table 2 [8].

**Table 1.** Estimates of deaths from AIDS and major co-infections with TB and fungal disease in 2015 at 12 months after diagnosis of infection, as a baseline for estimates. It is assumed that all untreated patients die of these infections, unless treated. ART, antiretroviral therapy; TB, tuberculosis; PCP, *Pneumocystis* pneumonia; CPA, chronic pulmonary aspergillosis.

cause of death	2015 estimate	basis of assumptions
all AIDS deaths	1 340 000	based on trend over past 5 years, extrapolated at a rate of 80 000 fewer per year
tuberculosis	400 000	380 000 in 2009, 350 000 in 2010, 430 000 in 2011, 320 000 in 2012, 360 000 in 2013, 400 000 in 2014
cryptococcal meningitis	232 756	baseline incidence of 75% of lowest Park (2009) estimate (278 250) [12], with a 5% screen and treat rate rising to 60% by 2020. Survival from screen and treat strategy is 75%, from fluconazole treatment of meningitis is 30% and amphotericin B and flucytosine treatment of meningitis is 60%
*Pneumocystis* pneumonia	260 034	number of patients with less than 200×10^6^ l^−1^ CD4 not on ART counts is 2 988 000 and 14.8% develop PCP each year (448 335). Assumed that 60% are treated and of these 70% survive
disseminated histoplasmosis	80 000	100 000 cases, of which 60% are not treated and survival in treated patients is 50%
chronic pulmonary aspergillosis complicating TB	56 288	23% of smear negative TB deaths are attributable to CPA (39 560) and 30% die of the 8.5% of pulmonary TB survivors who develop CPA

**Table 2.** Estimating of the proportion of pulmonary tuberculosis cases worldwide that are smear negative in HIV-infected patients, based on 2013 figures (WHO 2014 TB report [8]). n.a., not available.

TB cases	all	cases notified	pulmonary TB cases	total deaths	survived		
					total	smear positive	smear negative
HIV positive	1 100 000	n.a.	935 000	360 000	629 000	358 530	270 470
HIV negative	7 900 000	n.a.	6 715 000	1 100 000	5 780 000	3 294 600	2 485 400
total	9 000 000	5 719 753	7 650 000	1 460 000	6 409 000	3 653 130	2 755 870

Scenarios for each fungal disease were constructed to estimate avoidable deaths from 2016 to 2020, using linear annual assumptions of both improved access to key diagnostic tests and antifungal therapy. These scenarios also assume that the diagnostic tests are applied appropriately and that antifungal therapy is administered in a timely fashion, according to current guidelines. These numerical improvements in care are aspirational and arbitrary, and none exceed 90%; all are stated in the text and provided in the electronic supplementary material, table S1. Numbers in the text are rounded to the nearest 100 for ease of reading. Sensitivity analyses are not provided in the text to simplify the messages, but the spreadsheet provided in the electronic supplementary material (table S1) allows individual country, region and continent analyses to be done, with alternative assumptions.

## 3. Patients at risk of opportunistic fungal infections and death with AIDS

Of the estimated 36.9 million people with HIV infection, 22 million are not currently receiving ART [13]. The risk of acquiring any opportunistic fungal infection increases with declining CD4 cell counts, especially when below a threshold of 200 × 10^6^ l^−1^. This decline to less than 200 × 10^6^ l^−1^ cells occurs within 6–10.5 years from seroconversion in patients without therapy [14–17]. Assuming that CD4 counts of the 22 million untreated HIV-infected people are distributed uniformly, then about 2 988 000 are at increased risk of acquiring a life-threatening fungal infection.

Prior to the institution of the 90–90–90 target, the number of AIDS deaths was falling at about 80 000 annually, reaching approximately 1 500 000 in 2013. With the *current* rates of progress in rolling out ART, 940 000 AIDS deaths are expected in 2020.

## 4. Impact of 90–90–90

**Figure 1.**
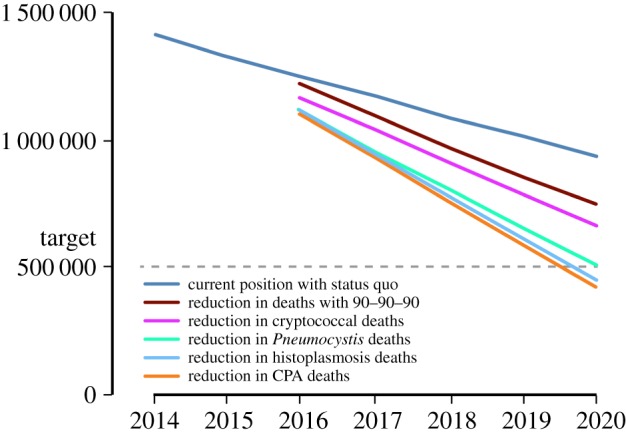
Reduction in AIDS deaths as depicted in the scenario described from 2014 to 2020. CPA, chronic pulmonary aspergillosis.

Gradual movement towards the 90–90–90 ART target will likely see a further reduction in deaths [18]. Many of those to be treated will have high CD4 counts and are not at risk of an early AIDS death. So an additional decline of 50% in deaths per year relative to current rates is estimated with improved ART coverage. This would add 200 000 annual survivors in 2020, and a cumulative total of lives saved between 2016 and 2020 of 600 000. However, even with this forecast, 740 000 people will still die of AIDS in 2020 (figure 1).

## 5. Cryptococcal disease

The most conservative estimate for cases of AIDS-related cryptococcal meningitis worldwide is 372 000 [12], calculated with 2007 data. Most patients affected are in their mid-30s and, untreated, the mortality is approximately 100%. Standard management is hospital-based and focused on antigen diagnosis, lumbar puncture and antifungal therapy. Assuming a 25% reduction in this already low baseline estimate, based on both improved ART coverage and early diagnosis in some countries, a conservative estimate is still 278 000 cases annually. In most high burden settings, antifungal therapy with fluconazole monotherapy is standard. Assuming that 60% of patients are diagnosed and treated with fluconazole and that the remainder remain undiagnosed and receive no treatment, one can expect an estimated 228 000 deaths annually (table 1). Currently, the 10-week mortality in fluconazole-treated cases is approximately 70% at 10 weeks [19,20].

There are broadly two approaches to reducing deaths from cryptococcal meningitis in AIDS. The first is based on detecting asymptomatic antigenaemia and oral treatment with fluconazole, after excluding meningitis (so-called ‘screen and treat’). Screen and treat in those with CD4 counts of less than 100 × 10^6^ l^−1^ reduces mortality from greater than 70% to 15–50% at 10 weeks, as long as meningitis is actively diagnosed and treated [20–22]. Here it is assumed that 40% of screened patients who were positive had meningitis [21,23]. The cost per life saved by screening has been estimated at $20–140, depending on the local incidence of disease [24]. The second approach to reducing mortality is improving treatment of established cases with amphotericin and flucytosine, as recommended by the WHO since 2013 [25]. Implementation of the WHO recommended treatment with amphotericin B and flucytosine should increase survival from approximately 30% with fluconazole to at least 60% [26–29]. The whole treatment cost of induction therapy for cryptococcal meningitis rises from approximately $150 with fluconazole monotherapy induction to approximately $450 with amphotericin B and flucytosine induction (followed by fluconazole consolidation therapy) [30,31].

Screen and treat programmes are operational in Rwanda and being rolled out in South Africa and several other African countries. If we assume 5% coverage currently, rising to 60% by 2020, then the number of identified early cases of cryptococcal antigenaemia would rise from 13 900 to 166 950 by 2020, all amenable to treatment. Deaths would fall from 232 756 to 166 394 cases, an annual saving in 2020 of 66 363 lives and a cumulative saving over the 5 years of 192 410 lives (electronic supplementary material, table S1).

With screen and treat, we can also assume improved diagnostic coverage for late presenting cases, as the same simple diagnostic test can be used. Thus, a direct benefit of rolling out the screen and treat programme is improved rapid diagnosis (estimated at 80% coverage by 2020) and a markedly reduced number of unscreened, undiagnosed patients who die—from 139 125 in 2015 to 55 650 patients in 2020. These figures are included with the screen and treat totals above.

For those patients who are not identified early by screen and treat, but present with meningitis, implementation of the WHO recommended treatment with amphotericin B and flucytosine should increase survival to approximately 70% at 10 weeks and 60% at 6 and 12 months [29]. If screen and treat were not rolled out, and only optimal antifungal therapy given, then deaths would fall about by 61 354 annually, assuming 60% coverage of optimal therapy, from an estimated 232 756 deaths in 2015 to 171 402 in 2020, a cumulative saving of 169 246 lives (electronic supplementary material, table S1).

The combination of both screen and treat (60% coverage) and optimal therapy (60% coverage) provides the maximal benefit, with annual deaths falling from an estimated 232 756 in 2015 to 162 876 in 2020, a cumulative gain of 300 554 lives (table 3 and figure 1).

**Table 3.** Estimated AIDS deaths over 5 years from 2016 to 2020 with 90–90–90 rollout, screening for cryptococcal disease, improved diagnosis and treatment of cryptococcal meningitis, *Pneumocystis* pneumonia, disseminated histoplasmosis and chronic pulmonary aspergillosis complicating pulmonary tuberculosis (TB). AIDS deaths in 2013 were estimated at 1 500 000 and in 2014, 1 420 000. The assumptions underlying this table are in the text and the electronic supplementary material. 90–90–90 = 90% of HIV-infected patients know their infection status, 90% of all HIV patients receiving ART and 90% viral load suppression; CPA, chronic pulmonary aspergillosis; TB, tuberculosis.

	2016	2017	2018	2019	2020	cumulative additional lives saved
AIDS deaths with status quo programmes	1 260 000	1 180 000	1 100 000	1 020 000	940 000	
reduction in AIDS deaths with 90–90–90	40 000	80 000	120 000	160 000	200 000	600 000
reduction in deaths with cryptococcal disease addressed	51 846	55 255	59 633	63 939	69 880	300 554
reduction in deaths with *Pneumocystis* pneumonia addressed	37 705	72 810	105 314	135 218	162 521	513 568
reduction in deaths with disseminated histoplasmosis addressed	8000	16 000	24 000	36 000	48 000	132 000
reduction in deaths with CPA complication of TB addressed	5629	11 258	19 701	25 329	33 773	95 689
reduction in deaths with 4 fungal diseases addressed	103 180	155 322	208 648	260 486	314 174	1 041 810
AIDS deaths if fungal diseases addressed and 90–90–90 rolled out	1 116 820	944 678	771 352	599 514	425 826	1 641 810

These estimates make the assumption that the incidence of asymptomatic cryptococcaemia and cryptococcal meningitis are constant over this 5-year period, which is unlikely. The incidence of cryptococcal infection is likely to fall as 90–90–90 rolls out. Some countries use extensive fluconazole prophylaxis, as this strategy antedated screen and treat, and this is also effective in preventing cryptococcal meningitis and deaths [32]. However, the potential gains estimated here in terms of survival are probably conservative overall, given the baseline assumptions used.

### (a) *Pneumocystis* pneumonia

It is not known how many patients with AIDS develop PCP. The incidence or point prevalence varies substantially, increasing as gross domestic product increases [33], from rates as low as 1.5% to as high as approximately 60%, partly depending on the population studied and diagnostic methods applied. Using a low median estimate of a 15% rate of PCP in those with fewer than 200 × 10^6^ l^−1^ CD4 cells [34–36], an estimated 450 000 develop PCP annually among the 2 988 000 with a CD4 count less than 200 × 10^6^ l^−1^. Those who default from ART or develop antiretroviral resistance are also at risk, but it is difficult to estimate this caseload, so 450 000 is probably a substantial underestimate.

All patients with PCP and AIDS who are not treated die. Diagnosis is usually clinical, but the presentation is often atypical, late and indistinguishable from bacterial pneumonia and pulmonary TB [37]. Molecular diagnosis from sputum, induced sputum or bronchoscopy fluid is the most sensitive and specific means of confirming the diagnosis; microscopy with silver or giemsa staining is about 75% sensitive, immunofluorescence microscopy about 90% compared with 95% for molecular diagnosis, but highly specific [38–44]. In the absence of bronchoscopy in children, induced sputum or nasopharyngeal aspiration with molecular detection is required [45,46]. Beta 1,3-d glucan is almost always detectable in the serum of patients with PCP, so can be used as a ‘rule-out’ test, but is non-specific if elevated [47]. Diagnosis with ‘typical’ clinical features tends to be late and carries a higher mortality than earlier, outpatient therapy. Treatment with high-dose cotrimoxazole (trimethoprim/sulfamethoxazole) is readily available throughout of the world.

Assuming that 60% of patients are treated and 70% of them survive, as is the case in most LMICs [45,48], the current annual mortality estimate from PCP is approximately 260 000 (table 1). If better diagnostics are instituted, with good clinical guidelines, the diagnosis and treatment rate should rise to 90%, and will include earlier diagnosis before hospitalization. If we assume that this improved diagnosis and treatment rate reduces deaths by 50% by 2020, then 238 750 lives will be saved that year (figure 1), and cumulatively more than 500 000 over 5 years. This estimation includes the assumption of an annual 5% fall in the rate of PCP attributable to combined rollout of 90–90–90 and prophylactic cotrimoxazole [49]. Unfortunately, neither cotrimoxazole prophylaxis nor ART are fully protective [50–53], so PCP will continue to occur despite excellent ART adoption, and HIV units need to able to diagnose and treat it.

Additionally, there is a strong case for improving the diagnosis of PCP because use of high-dose cotrimoxazole carries much toxicity. Adverse reactions of high-dose cotrimoxazole include nausea and vomiting (90%), reduced oral intake in already malnourished patients, neutropenia (50%), pruritic rashes (33%), elevated liver function tests (33%) and significant anaemia (20%) [54,55]. A negative molecular test on a good-quality specimen virtually rules out the diagnosis of PCP and unnecessary exposure to harm can be avoided.

### (b) Disseminated histoplasmosis

The number of DH cases in AIDS has been estimated to be between 100 000 and 300 000 [56,57]. Very high rates are confined to certain countries and localities. For example in Fortaleza, Brazil, 164 (43%) of 378 consecutively hospitalized HIV patients had DH [58] and in Venezuela, autopsies of patients with AIDS revealed histoplasmosis in 29 of 66 (44%) [59]. These focal hotspots make global estimations of burden challenging, especially as only one study has addressed the frequency of DH in AIDS in Africa [60], whereas histoplasmosis has been documented across the continent for over 40 years [61].

The fungus that causes DH is *Histoplasma capsulatum*, a slow growing, small, intracellular yeast that enters the body via inhalation. Bat guano is its primary ecological niche, and there is substantial seasonal variation in case numbers. The clinical presentation of DH is subtly different from TB in patients with AIDS, with more gastrointestinal and fewer respiratory features, pyrexia and usually some degree of pancytopenia, also seen as part of HIV infection itself. Most patients with DH in the context of AIDS are in their 30s, and death without therapy usually occurs in 10–14 days. The fungus typically takes two weeks to grow on mycological media and does not grow on media used for bacterial culture. Patients with CD4 counts < 200 × 10^6^ l^−1^ are at most risk [62]. Rapid diagnosis can be achieved by careful examination of a blood film (40% sensitivity), bone marrow examination (more than 90% sensitivity), antigen detection in serum or urine (70–90% sensitivity) [63] and molecular methods on blood (more than 95% sensitivity). Greatly improved detection rates and reduced mortality have been shown with use of either antigen detection or molecular methods [64,65].

The antifungal agents of choice for DH are amphotericin B and itraconazole; fluconazole is not effective for DH in AIDS. Improved outcomes with early diagnosis and appropriate therapy can achieve 85% survival [66,67].

Taking the most conservative estimate of annual incidence, of 100 000 cases and assuming that the diagnosis is only made in 40% of patients currently with a 50% survival in those treated, including relapse deaths [68,69], we would anticipate about 80 000 annual deaths attributable to DH. In centres without rapid diagnosis, but a high awareness of DH, current mortality rates are more than 45% [64,70], whereas those diagnosed rapidly have a mortality of less than 30% [62,71].

If antigen and/or PCR were made available in all high incidence communities in the Americas (assumed to be approx. 70% global coverage), and both itraconazole and amphotericin B were also used, we would anticipate a reduction of approximately 60% of DH deaths by 2020. This translates into a survival gain of 48 000 lives annually by 2020 (figure 1), and cumulatively 132 000 lives over 5 years.

### (c) Chronic pulmonary aspergillosis complicating tuberculosis in HIV-positive patients

CPA is a progressive and usually fatal complication of multiple pulmonary disorders, notably TB, chronic obstructive pulmonary disease and those with non-tuberculous mycobacterial infections [72]. Its radiological and clinical manifestations are similar to TB, although fever is uncommon and it is often mistaken for smear-negative TB. The detection in serum of *Aspergillus fumigatus* IgG is the key diagnostic test, [73] but is barely available in LMICs. Treatment options are amphotericin B or itraconazole, or the newer azoles and echinocandins, and are approximately 60% effective [72,73]. ART rollout is unlikely to reduce the incidence of CPA as it is an infection of non-immunocompromised patients, but reduction in pulmonary TB cases will.

About 935 000 patients developed pulmonary TB in the context of HIV infection in 2013 (table 2). Of these, approximately 360 000 died and 73% of these deaths occurred in the African region. In all pulmonary TB patients diagnosed in 2012 whose sputum was analysed by microscopy, 1 900 000 of 4 400 000 (43%) were smear negative [8]; of the 400 000 deaths, approximately 172 000 were, therefore, in smear-negative patients. Later culture confirmation of TB is expected in approximately 19–39% of these patients [74,75], leaving 104 900–139 300 (mean 122 000) without confirmed TB. In an unpublished study of 39 HIV positive, smear-negative patients in Kampala with very low CD4 counts, 26% had elevated *Aspergillus* IgG antibodies and 40% of these patients died within two months [76]. In HIV-negative patients, the *Aspergillus* IgG antibody detection method used in this Kampala study had a 96% sensitivity and 98% specificity for CPA [77]. We have, therefore, conservatively estimated that 26% of these 172 000 (40 248) deaths are attributable to CPA, as false negatives are likely in this highly immunocompromised group.

In addition, there were 255 850 HIV-infected survivors of ‘smear-negative’ TB in 2013. Another prospective study showed that 2–7 years after TB treatment, 8.5% of Ugandan patients had CPA, whether HIV infected or not [78]. This is a conservative rate, as detectable *Aspergillus* antibodies were found in 34% of post-TB patients in UK in the 1960s, and 30% of those during treatment for pulmonary TB in Iran [79,80]. Assuming that 8.5% of the annual 595 000 survivors of TB (both smear-positive and negative) develop CPA in 1 year, 53 645 would be affected. However, the chronicity of CPA certainly underestimates the magnitude of this problem. Figures from older, non-HIV-infected patient series from Korea and Japan puts the first-year mortality after presentation at approximately 30% [81]. These two figures combined yield a conservative mortality estimate of more than 56 250 HIV-infected patients dying of CPA annually.

If the diagnosis and treatment of CPA are enabled by provision of *Aspergillus* IgG testing, and availability of itraconazole and amphotericin B treatments, for 60% of patients, and is 60% effective (i.e. mortality reduction to 12%), then mortality should fall to 20 600 (figure 1). If the rollout of diagnosis and therapy improves from an arbitrary low baseline of 5% to 60% by 2020, then the cumulative lives saved would be at least 87 800.

### (d) Actions required to improve outcomes

Early diagnosis and treatment of the most common fungal diseases are important steps in preventing AIDS-related deaths. The actions described in table 4 would have a major impact on survival, especially if undertaken widely across high and middle HIV burden countries. A disease systems analysis from Tanzania highlighted the failure to address opportunistic infections as a major cause of mortality [82]. The predictive scenarios are illustrative of the potential impact of targeting fungal diseases in AIDS and cannot be definitive, given the poverty of accurate data on incidence for most communities. ART rollout through 90–90–90 is important, but insufficient to reduce deaths. Progress will be incomplete because ART coverage will not be 100% and ART reduces the incidence of most co-infections but not all.

**Table 4.** Key public health actions required to improve the outcome from cryptococcal disease, *Pneumocystis* pneumonia, disseminated histoplasmosis and chronic pulmonary aspergillosis complicating tuberculosis, all in HIV-infected patients. WHO, World Health Organization; CSF, cerebrospinal fluid; LFA, lateral flow assay; BAL, bronchoalveolar lavage; AFB, acid fast bacilli; LMIC, low- and middle-income countries; NPA, nasopharyngeal aspirate; PCR, polymerase chain reaction; PAHO, Pan American Health Organization; TB, tuberculosis; EML, essential medicine list.

disease entity	diagnostic test	diagnostic action	treatment	proposed action to be taken
cryptococcal antigenaemia pre-meningitis	antigen test on serum, plasma and whole blood	screening <200 (<100) CD4 counts	fluconazole therapy	promote rapid adoption of WHO guidelines ensure access to rapid antigen tests
cryptococcal meningitis	antigen test on blood or CSF, lumbar punctures	rapid antigen testing (LFA) lumbar punctures	amphotericin B + flucytosine, followed by fluconazole	promote rapid adoption of WHO guidelines ensure access to rapid antigen tests improve access to drugs in high burden countries
*Pneumocystis* pneumonia	microscopy or PCR on sputum, induced sputum or BAL, NPA PCR in children	enable rapid testing on respiratory samples on AFB smear-negative samples	empirical therapy usually given, so discontinuation of unnecessary empirical therapy in those negative oral therapy of mild cases avoiding hospitalization	promote implementation of rapid molecular diagnostic develop treatment guidelines for LMICs
disseminated histoplasmosis	antigen test on serum or urine PCR on blood	antigen (or PCR) testing on hospitalized HIV patients in relevant countries	immediate treatment with amphotericin B, followed by itraconazole. Itraconazole alone for mild cases, avoiding hospitalization	ensure access to antigen and/or PCR diagnosis and drugs in high burden countries develop WHO/PAHO treatment guidelines. Place itraconazole on the WHO EML and national EMLs
chronic pulmonary aspergillosis in ‘smear-negative TB’	serum *Aspergillus* IgG antibody test	testing all AFB smear-negative cases, and any relapse cases	itraconazole or amphotericin B	ensure access to *Aspergillus* antibody diagnosis and drugs in high burden countries place itraconazole on the WHO EML and national EMLs

The 2013 WHO cryptococcal disease guidelines [25] recommend antigen screening for early detection of asymptomatic antigenaemia with the low-cost point of care lateral flow assay, and treatment with amphotericin B and flucytosine for meningitis. Only some LMICs have established screening for cryptococcal antigen in those with low CD4 cell counts, a critically important intervention. The Diflucan Partnership Programme funded by Pfizer provides fluconazole for established disease, not prevention of meningitis in those with asymptomatic antigenaemia, so alternative sources and funding are required. Amphotericin B and/or flucytosine are not available in many countries owing to various constraints including lack of registration, despite being on the WHO Essential Medicines List (EML) [83].

PCP is a major AIDS-defining illness. Diagnosis is usually empirical and based on severity and sometimes obvious, but late clinical features. Concurrent bacterial infection with PCP is common [84,85], and some deaths attributed to bacterial pneumonia may mask PCP cases. Microscopy is simple and inexpensive, and should be more widely used (see www.microfungi.net). Many molecular diagnostic tests are available but require a frozen transport chain and reliable electricity and are also expensive. Diagnosis with the β 1,3-d glucan test as a rule-out test will not be feasible for many settings owing to high costs of this assay. A low-cost diagnostic test is required for LMICs. While the best therapy, co-trimoxazole, is almost universally available, and the WHO has issued guidelines on its use for prophylaxis, there are no WHO guidelines on treatment of PCP, although the American Thoracic Society has issued management recommendations [86]. There is a need for clear guidelines for LMICs including recommendation on the use of corticosteroids for presumptive PCP in high TB burden countries.

The annual incidence of histoplasmosis is not known in many countries in which it is endemic. This is a barrier to adoption of the commercially available rapid ELISA-based antigen assays now commercially available, and simple low-cost lateral flow assay that will be available soon. Despite its importance in the treatment of histoplasmosis, itraconazole is not on the WHO EML, affecting availability in many countries. Similar to PCP neither the WHO nor Pan American Health Organization (PAHO), where the largest burden of disease falls, have developed guidelines for the treatment of histoplasmosis; publication and dissemination would improve both diagnosis and treatment and prevent deaths.

The annual incidence and 5-year point prevalence of CPA after TB were estimated using 2007 TB data and published in 2011; 372 385 incident cases per year and a prevalence of 1 173 881 (range 852 048–1 372 457) patients affected [87]. At that time there were no data related to frequency and diagnosis of CPA in HIV-infected patients. Since then, a prospective survey in HIV-positive and -negative patients in Uganda has been completed; both groups developed CPA at the same frequency after pulmonary TB and raised *Aspergillus* antibodies are found in both groups [78]. Therefore, it is reasonable to assume similar rates of CPA in HIV-positive and -negative patients. Furthermore, a high frequency (26%) of raised *Aspergillus* antibodies and a two-month 40% mortality were found in a group of hospitalized smear- and mycobacterial culture-negative patients in Kampala, clearly indicating that many HIV patients have pulmonary aspergillosis. Whether these cases are strictly CPA or represent invasive aspergillosis [88,89] is conjecture, but in either case they are potentially responsive to antifungal therapy, if diagnosed early enough. Diagnosis of CPA requires radiology and *Aspergillus* IgG antibody detection. Fungal cultures for *Aspergillus* spp. are insensitive for CPA, not specific and may be laboratory contaminants. Recent European guidelines on the diagnosis and management of CPA are helpful [72], but additional research on the frequency of CPA, when TB patients should be tested and the best interventions in HIV-infected patients are all required.

These estimates of lives saved ignore other potentially fatal but treatable fungal diseases in AIDS, including oesophageal and invasive candidiasis [89,90], paracoccidioidomycosis [91], coccidioidomycosis [91,92], blastomycosis [93] and *Talaromyces* (*Penicillium*) *marneffei* infection [94,95]. Enhancing capability to diagnose fungal diseases worldwide is required [57].

## 6. Conclusion and recommendation

Scenario modelling and limited epidemiological data strongly suggest that the annual 700 000 AIDS deaths related to the four most common lethal fungal infections could be significantly reduced by the wide adoption of current diagnostic tests, antifungal agents and known systematic strategies. As the true burden of lethal fungal infections is not known, these estimates are probably conservative, although the recent (2014) UNAIDS revision downwards of AIDS deaths to 1 200 000 (from 1 500 000 in 2013) will likely alter the precise estimates, but not the thrust of the arguments.

Depending on implementation and uptake, over 300 000 deaths might be saved annually, in addition to those saved by ART rollout, bringing total deaths down to 426 000, well below 500 000, by 2020 and lower subsequently, aiming to achieve the Millennium Development Goal for 2030 of ‘ending AIDS’. Preventing deaths from AIDS represents excellent value for money in a medical and broad societal sense as it prevents the loss of the most economically active participants in society. These lives can be saved in a manner that avoids duplication of efforts, by integrating fungal disease interventions and treatment guidelines into existing HIV and TB programmes and national strategies.

## Supplementary Material

Modelling reduction in AIDS deaths Dec 2015

## Endnote

1 90–90–90 = 90% of HIV-infected patients know their infection status, 90% of all HIV patients receiving ART and 90% viral load suppression.
